# Identifying a High Fraction of the Human Genome to be under Selective Constraint Using GERP++

**DOI:** 10.1371/journal.pcbi.1001025

**Published:** 2010-12-02

**Authors:** Eugene V. Davydov, David L. Goode, Marina Sirota, Gregory M. Cooper, Arend Sidow, Serafim Batzoglou

**Affiliations:** 1 Department of Computer Science, Stanford University, Stanford, California, United States of America; 2 Department of Genetics, Stanford University School of Medicine, Stanford, California, United States of America; 3 Biomedical Informatics Program, Stanford University, Stanford, California, United States of America; 4 Department of Genome Sciences, University of Washington, Seattle, Washington, United States of America; 5 Howard Hughes Medical Institute, University of Washington, Seattle, Washington, United States of America; 6 Department of Pathology, Stanford University School of Medicine, Stanford, California, United States of America; University of British Columbia, Canada

## Abstract

Computational efforts to identify functional elements within genomes leverage comparative sequence information by looking for regions that exhibit evidence of selective constraint. One way of detecting constrained elements is to follow a bottom-up approach by computing constraint scores for individual positions of a multiple alignment and then defining constrained elements as segments of contiguous, highly scoring nucleotide positions. Here we present GERP++, a new tool that uses maximum likelihood evolutionary rate estimation for position-specific scoring and, in contrast to previous bottom-up methods, a novel dynamic programming approach to subsequently define constrained elements. GERP++ evaluates a richer set of candidate element breakpoints and ranks them based on statistical significance, eliminating the need for biased heuristic extension techniques. Using GERP++ we identify over 1.3 million constrained elements spanning over 7% of the human genome. We predict a higher fraction than earlier estimates largely due to the annotation of longer constrained elements, which improves one to one correspondence between predicted elements with known functional sequences. GERP++ is an efficient and effective tool to provide both nucleotide- and element-level constraint scores within deep multiple sequence alignments.

## Author Summary

There are millions of sequences in the human genome that perform essential functions, such as protein-coding exons, noncoding RNAs, and regulatory sequences that control the transcription of genes. However, these functional sequences are embedded in a background of DNA that serves no discernible function. Thus, a major challenge in the field of genomics is the accurate identification of functional sequences in the human genome. One approach to identify functional sequences is to align the genome sequences of many divergent species and search for sequences whose similarity has been maintained during evolution. We have developed GERP++, a software tool that utilizes this “comparative genomics” approach to identify putatively functional sequences. Given a multiple sequence alignment, GERP++ identifies sites under evolutionary constraint, i.e., sites that show fewer substitutions than would be expected to occur during neutral evolution. GERP++ then aggregates these sites into longer, potentially functional sequences called constrained elements. Using GERP++ results in improved resolution of functional sequence elements in the human genome and reveals that a higher proportion of the human genome is under evolutionary constraint (∼7%) than was previously estimated.

## Introduction

The identification and annotation of all functional elements in the human genome is one of the main goals of contemporary genetics in general, and the ENCODE project in particular [1], [2], [3]. Comparative sequence analysis, enabled by multiple sequence alignments of the human genome to dozens of mammalian species, has become a powerful tool in the pursuit of this goal, as sequence conservation due to negative selection is often a strong signal of biological function. After constructing a multiple sequence alignment, one can quantify evolutionary rates at the level of individual positions and identify segments of the alignment that show significantly elevated levels of conservation.

Several computational methods for constrained element (CE) detection have been developed, with most falling into one of two broad categories: generative model-based approaches, which attempt to explicitly model the quantity and distribution of constraint within an alignment, and bottom-up approaches, which first estimate constraint at individual positions and then look for clusters of highly constrained positions. A widely used generative approach, phastCons [4], uses a phylo-Hidden Markov Model (HMM) to find the most likely parse of the alignment into constrained and neutral hidden states. While HMMs are widely used in modeling biological sequences, they have known drawbacks: transition probabilities imply a specific geometric state duration distribution, which in the context of phastCons means predicted constrained and neutral segment length. This may bias the resulting estimates of element length and total genomic fraction under constraint.

One of the leading bottom-up approaches is GERP [5], which quantifies position-specific constraint in terms of rejected substitutions (RS), the difference between the neutral rate of substitution and the observed rate as estimated by maximum likelihood, and heuristically extends contiguous segments of constrained positions (RS>0) in a BLAST-like [6] manner. However, GERP is computationally slow because its maximum likelihood computation uses the Expectation Maximization algorithm [7] to estimate a new set of branch lengths for each position of the alignment; this step is also undesirable methodologically because it involves estimating k real-valued parameters from k nucleotides of data. Furthermore, the extension heuristic used by GERP (and other bottom-up methods [8]) may induce biases in the length of predicted CEs.

In this work we present GERP++, a novel bottom-up method for constrained element detection that like GERP uses rejected substitutions as a metric of constraint. GERP++ uses a significantly faster and more statistically robust maximum likelihood estimation procedure to compute expected rates of evolution that results in a more than 100-fold reduction in computation time. In addition, we introduce a novel criterion of grouping constrained positions into constrained elements using statistical significance as a guide and assigning p-values to our predictions. We apply a dynamic programming approach to globally predict a set of constrained elements ranked by their p-values and a concomitant false positive rate estimate. Using GERP++ we analyzed an alignment of the human genome and 33 other mammalian species, identifying over 1.3 million constrained elements spanning over 7% of the human genome with high confidence. Compared to previous methods, we predict a larger fraction of the human genome to be contained in constrained elements due to the annotation of many fewer but longer elements, with a very low false positive rate.

## Results

### Overview of Algorithm

**Figure 1:**
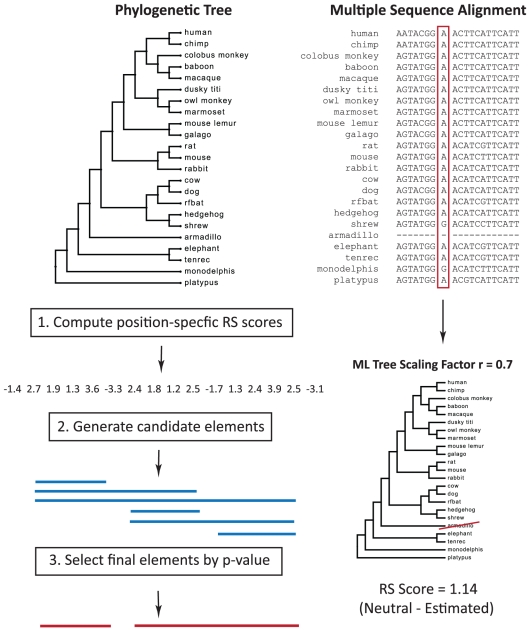
Overview of GERP++. (1) For each position of the multiple alignment we compute the conservation score in rejected substitutions by subtracting the estimated evolutionary rate from the neutral rate. The neutral rate is computed by removing species gapped at that position from the phylogenetic tree and summing the branch lengths of the resulting projected tree; the evolutionary rate is estimated by computing the maximum likelihood rescaling of the projected tree. (2) Given position-specific conservation scores, we generate a set of candidate elements. (3) For each candidate element, we compute a p-value to represent the likelihood of observing a segment of equal length and greater than or equal score under the null model. We then select a non-overlapping set of elements in order of increasing p-value.

Like other bottom-up approaches, the GERP++ algorithm consists of two components: calculation of position-specific constraint scores for each column of a multiple alignment, and subsequent aggregation of neighboring columns into segments that score significantly higher than expected by chance (Fig 1; see Methods for more detailed description). These are largely independent procedures: the GERP++ score for a specific position depends entirely on the nucleotides at that position and not on any global element predictions, while identification of statistically significant high-scoring segments depends only on the additivity of individual position scores and can potentially be used in conjunction with other position-specific scoring metrics.

Constraint intensity at individual alignment positions is quantified in terms of “rejected substitutions” (RS), defined as the number of substitutions expected under neutrality minus the number of substitutions “observed” at the position [5]. Thus, positive scores represent a substitution deficit (which would be expected for sites under selective constraint), while negative scores represent a substitution surplus. To estimate this quantity at each aligned position, GERP++ begins with a pre-defined neutral tree relating the genomes present within the alignment that supplies both the total neutral rate across the entire tree and the relative length of each individual branch. For each alignment column, we estimate a scaling factor, applied uniformly to all branches of the tree, that maximizes the probability of the observed nucleotides in the alignment column. The product of the scaling factor and the neutral rate defines the ‘observed’ rate of evolution at each position.

Then, in the element-finding step, GERP++ uses the position-specific RS scores to generate a set of candidate elements. For each putative element it computes a p-value based on the element's length and score (defined as the sum of RS scores for each position within the element) that represents the probability of observing such an element in the null model. These p-values are used to rank CEs in order of significance and report a set of non-overlapping predictions, starting with the lowest (best) p-value. Rather than applying a fixed cutoff, GERP++ estimates the false positive rate by randomly permuting the input RS-scores and treating any prediction within the shuffled sequence as a false positive, similar to the first version of GERP [1], [5].

### Constraint in the Human Genome

We used GERP++ to analyze the TBA alignment of the human genome to 33 other mammalian species (the most distant mammalian species is Platypus) spanning over 3 billion positions with a phylogenetic scope of 5.83 substitutions per neutral site. We identified 1,354,034 constrained elements covering 214,749,502 nucleotides, or approximately 7% of the human genome, with an estimated false positive rate of 0.86% at the nucleotide level (see Methods for details). Compared to a slightly negative background average of −0.125 RS, GERP++ predictions and certain known functional elements display an elevated level of constraint, in excess of 1.7 RS. GERP++ elements range in size from 4 to nearly 2000 bases, with mean length of 158.6 nucleotides. The minimum (4 bases) and maximum lengths (2000 bases) are parameters of the algorithm, and the tail of the length distribution (Fig S2A) suggests that with a more permissive upper bound even longer elements could be identified.

**Figure 2:**
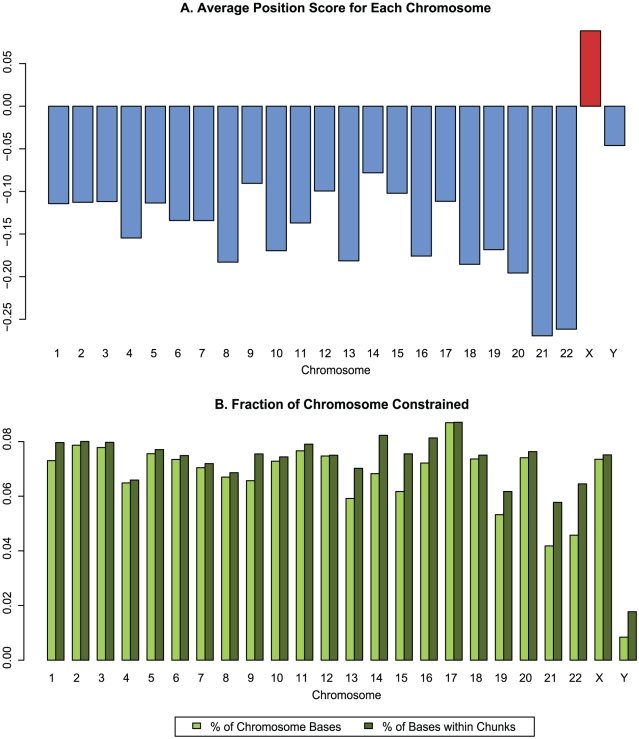
Per-chromosome constraint intensity. (A) Mean RS score for all alignment positions where evolutionary rate was computed. Note the elevated average score for chromosome X. (B) Fraction of chromosome that falls into predicted constrained elements. Light green bars show fraction of entire chromosome, while dark green bars show fraction adjusted for regions where no rate computation was performed and no elements could span (see Methods).

We observe significant variation among entire chromosomes of both average RS score and fraction of positions predicted to belong to constrained elements (Fig 2). The mean constraint level varied from −0.3 to −0.05 RS with the exception of chromosome X, which was the only chromosome with a positive average RS score, just under 0.1 RS. This result is consistent with earlier work [9], which suggested that the X chromosome in rodents has a reduced mutation rate. We also observe substantial fluctuation in the fraction of each chromosome predicted to be inside constrained elements, which varied from 1% of the Y chromosome to 4–9% for other chromosomes. We expect this metric to be low for the Y chromosome because a large portion of the alignments for the Y chromosome are too shallow to perform a rate estimation, but even when adjusting for “effective” chromosome size much of the fluctuation remains (Fig 2B). Surprisingly, despite a low fraction of the Y chromosome being within constrained elements, it does not have a particularly low average RS score, while the X chromosome does not exhibit a high CE fraction despite the positive average RS. In fact, there appears to be at best weak correlation between these two metrics of constraint: since the null model is derived from the actual distribution of RS scores for a given region, any (additive) difference in RS score applied uniformly to every position in the region would not change the p-value of any candidate element (although in practice this would alter the exact boundaries, resulting in a slightly different candidate set). The chromosomal fraction within predicted constrained elements ultimately depends more on the distribution and variance of the scores rather than the mean. Unfortunately, this is impossible to quantify exactly due to confounding factors such as differences in alignment quality and depth.

### Estimating Detectable Constraint

The only major parameter for GERP++ is a false positive rate cutoff that determines at what point the algorithm should stop generating predictions in order to avoid too many false discoveries. Throughout its execution GERP++ keeps track of the constrained elements predicted so far, as well as estimates of the number and total size of false positive predictions for the specified cutoff level. Examining how these quantities grow as the cutoff parameter increases permits us to estimate the amount of total constraint that can be detected using this methodology and give an approximate upper bound on the amount of constraint within the human genome.

**Figure 3:**
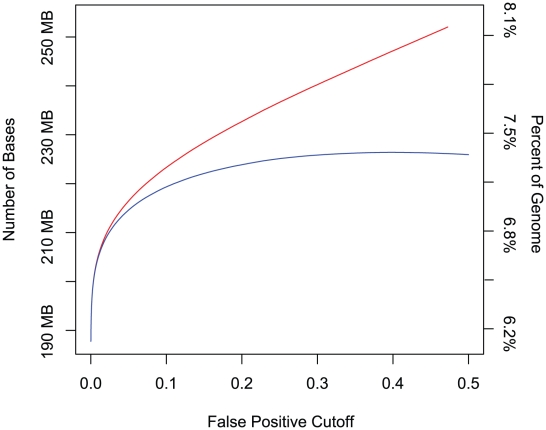
Estimating detectable constraint. The red curve represents the number of bases within predicted constrained element as a function of the false positive cutoff parameter. The blue curve represents the number of predicted bases minus the expected number of false positive bases, also as a function of the false positive cutoff.

Let B(*c*) be the number of bases within constrained elements predicted at false positive cutoff *c*, and let B^*^(*c*) = B(*c*)−F(*c*) be the same quantity adjusted for false positive predictions by subtracting the estimated number of false positive bases (as found in shuffled alignments) at cutoff *c*. Fig 3 shows B and B^*^ as a function of *c* from 0 to 50%: while B continues to increase, B* starts to level off right as B begins to grow linearly. This suggests that max*_c_* B^*^(*c*) can be used to estimate the total number of bases in constrained elements that can be annotated using this method in any given region or the entire genome. Approximately 225 megabases, or nearly 7.3% of the human genome can be detected as contained in CEs using GERP++ at the mammalian phylogenetic scope. If we adjust for the portions of the genome where rate estimation was not performed (but with a deeper alignment might be in the future), we estimate that up to 8% of the human genome consists of CEs detectable using this kind of methodology. Combined with the observation that about 190 megabases, or 6.2% can be detected at a false positive cutoff of 0 (Fig 3), we obtain a fairly narrow estimate of 6–8% of the human genome under detectable evolutionary constraint, in the mammalian scope. We note that this estimate depends on alignment quality, since we may fail to pinpoint constrained elements not only due to method-intrinsic limitations but also because an appropriate signal may be absent in a given multiple alignment.

### Association of Predicted CEs with Known Functional Elements

**Figure 4:**
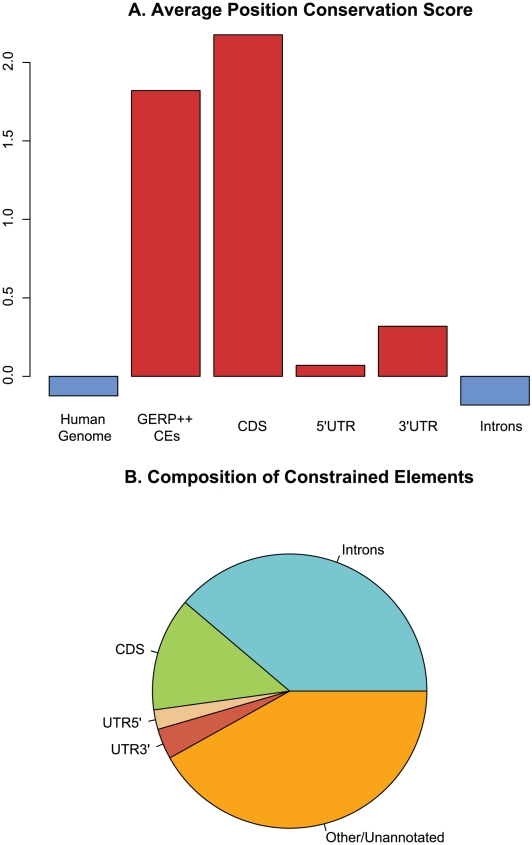
Relationship between CEs and known functional elements. (A) Mean rejected substitution scores for entire human genome, constrained elements predicted by GERP++, and known annotated exons, introns, and UTR regions. (B) Breakdown of constrained element positions by region type.

We next examine the relationship between evolutionary constraint and several classes of biologically important regions. Overall, coding exons exhibit by far the strongest levels of constraint, as quantified both by the average RS score within functional elements (Fig 4A), and by fraction of bases that overlap the predicted CEs (see Table 1). Both 5′ and 3′ UTR regions show weaker but noticeable constraint levels and, somewhat surprisingly, introns on average have slightly lower RS scores than the overall genomic baseline. However, a nontrivial fraction of introns does exhibit evidence of constraint, as nearly 7% of intron positions overlap predicted elements (Table 1), and these positions make up a large fraction of constrained element bases (see Fig 4B).

**Table 1:** Fraction of functional regions covered by constrained elements on a nucleotide level.

Annotation	% Coverage by CEs
Exons	84.6%
Introns	6.9%
UTR5′	23.7%
UTR3′	33.9%
ncRNA	10.1%

Over 94% of the coding exons in the human genome overlap at least one predicted CE; conversely, only about 16% of constrained elements overlap a coding exon. CEs that overlap exons are on average ∼60 nucleotides or 40% longer, and consequently have more than two-fold higher scores, than elements that do not overlap exons (both t-tests significant at p-value<2.2·10−16). While overall these results are consistent with what was observed using the previous version of GERP [5] on much more limited alignments, the length difference between exon-associated and non-overlapping CEs is somewhat smaller than what was previously found. This is partially explained by the differences in the pattern of constraint between coding exons and other regions. Because the previous GERP by default only merges blocks of contiguous constrained positions if they are separated by at most one unconstrained position [5], it is far more likely to generate longer elements in exonic regions where most unconstrained bases correspond to 3rd positions of a codon and are usually flanked by constrained positions. In noncoding regions where unconstrained positions are distributed more irregularly and often occur consecutively, the previous GERP algorithm [5] ends up fragmenting longer constrained regions and generating shorter elements. Because GERP++ does not base merging decisions on any such fixed threshold it is able to better annotate longer noncoding CEs.

**Figure 5:**
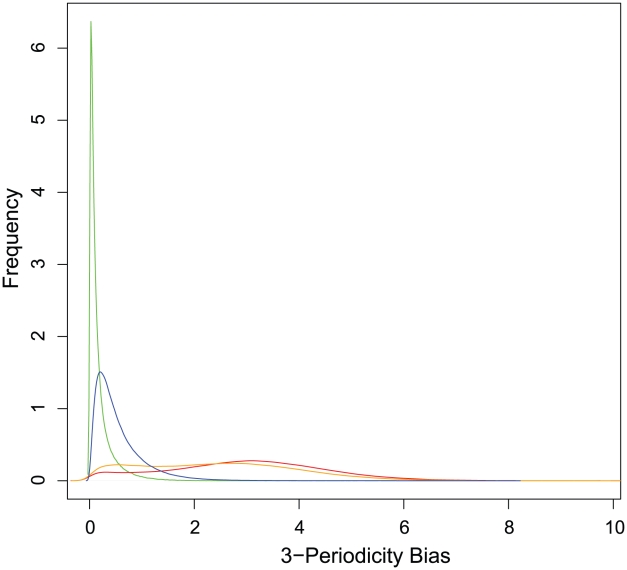
Distributions (smoothed histograms) of 3-periodicity bias for known exons (red), introns (green), CEs that overlap exons (orange), and CEs not overlapping exons (blue).

To further test this hypothesis, and to investigate a potentially useful signal for detecting coding exons, we introduce a metric that rigorously quantifies this pattern of constraint for any region. For any given segment, we define the 3-periodicity bias as the maximum over the 3 possible reading frames of the mean RS score at positions 1 and 2 minus the mean RS score at position 3. This metric quantifies a periodic bias in constraint and effectively deals with unknown reading frame location and lack of a reading frame altogether, since the maximum is taken over all 3 possibilities. As Fig 5 shows, the 3-periodicity bias is a strong signal characteristic of coding exons (mean 2.96) compared to other regions such as UTRs, introns, and ncRNAs (mean 0.13–0.38, difference significant at p-value<2.2·10^−16^). We partitioned the constrained elements predicted by GERP++ according to exon overlap, and found that CEs overlapping coding exons have a much greater mean 3-periodicity bias (Table 2). However, the difference between CEs that did not overlap any annotated exons, and known nonexonic regions such as introns was still significant, suggesting some of these CEs intersect unannotated exonic regions. To test this hypothesis, we checked the constrained elements that did not overlap any known coding exons against exon predictions made by the computational gene prediction tool CONTRAST [10]. We found 16,881 CEs (making up 1.5% of all CEs that did not overlap known genes) that overlapped CONTRAST predictions, and these CEs had a significantly higher 3-periodicity bias (1.33) than those that did not overlap CONTRAST predictions (0.54). As this latter figure is still higher than the average 3-periodicity of clearly non-exonic elements, it is possible that a fraction of these elements overlap unannotated exons or pseudogenes with recently lost function. It is interesting to note that the difference between 3-periodicity bias of GERP++ CEs that overlap known exons (2.46) and CEs that overlap CONTRAST predictions (1.33) is also significant. This is likely a combination of two factors: false positives (or errors in identifying the exact boundary) in CONTRAST predictions, and selection bias that manifests as exons with higher 3-periodicity being more conserved and/or easier to identify, and thus annotated in the UCSC Known Genes set.

**Table 2:** Mean 3-periodicity bias for different types of regions.

Type	Mean 3-periodicity Bias
Exons	2.96
5′ UTR	0.57
3′ UTR	0.32
Introns	0.18
CEs overlapping exons	2.46
CEs not overlapping exons	0.55

### Comparison with PhastCons

**Figure 6:**
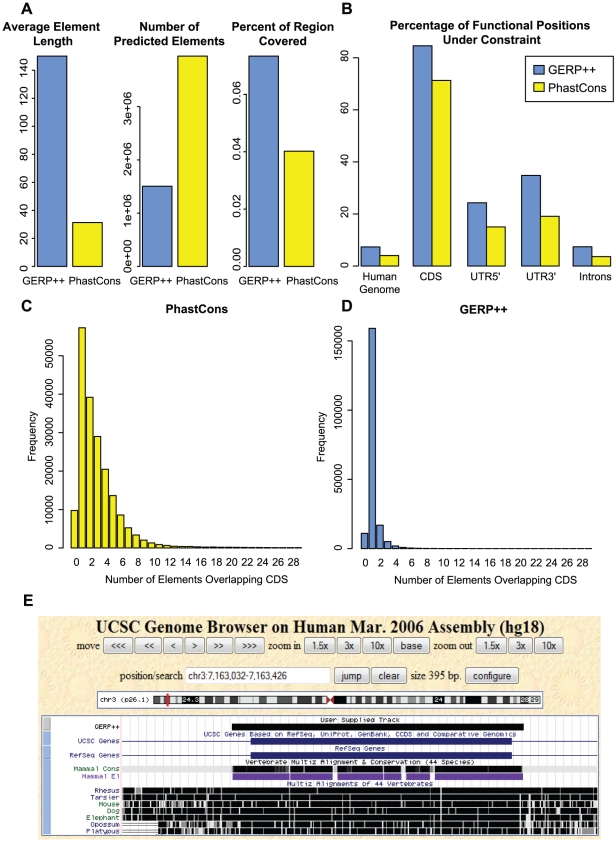
GERP++ vs phastCons predictions. (A) Mean length (left), number (middle) and total length (right) of constrained elements predicted by GERP++ (blue) and phastCons(yellow). (B) Nucleotide-level fraction of annotated exons, introns, UTRs and noncoding RNAs genes covered by GERP++ (blue) and phastCons (yellow) predictions. (C&D) Histogram of number of distinct predicted GERP++ (blue, D) and phastCons(yellow, C) constrained elements overlapping each annotated coding exon. Note the difference in scale on the y-axis. (E) A constrained region slightly over 200 base pairs in length that contains a known exon, as annotated by GERP++ (labeled ‘GERP++’, black) and phastCons (purple track labeled ‘Mammal El’). Note how phastCons fragments the exon into multiple CE predictions.

We compared the GERP++ constrained element predictions in placental mammals (see Methods) to phastCons [4], the leading generative model-based tool. Not surprisingly, we found significant overlap between GERP++ and phastCons predictions: 80% of GERP++ predictions overlapped at least one phastCons prediction, and vice versa. However, aside from both algorithms detecting clearly constrained areas, there are substantial differences: GERP++ predicts significantly fewer elements, which are much longer on average (see Fig S2B for distribution of phastCons element lengths) and cover a substantially larger portion of the human genome - almost twice as much as the 4% predicted by phastCons (Fig 6A). As a result, on a nucleotide level GERP++ overlaps 90% of phastCons predictions while only half of GERP++ CE positions are covered by phastCons.

**Figure 7:**
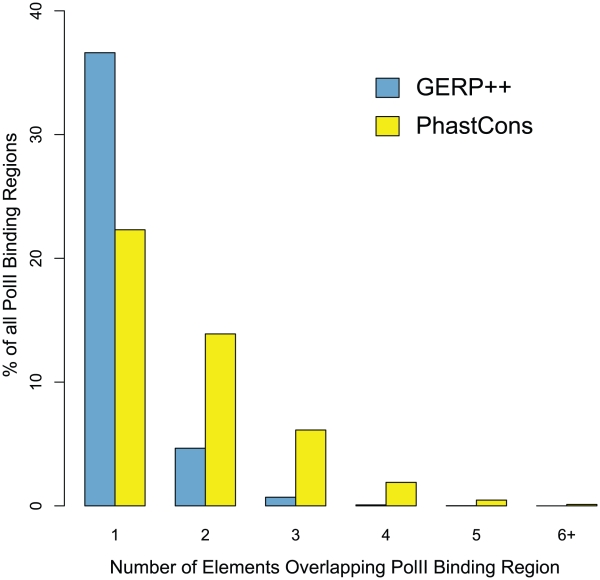
Mean distribution of PolII binding sites by number of overlapping CEs over 9 Encode PolII ChIP experiments, for GERP++ and phastCons.

Part of the reason for these differences is that often phastCons predicts multiple elements where GERP++ makes one longer prediction. PhastCons thus skips intermediate positions which may be under weaker constraint yet still part of one large functional element, as the example in Fig 6E shows. In order to demonstrate that this is not an isolated occurrence and to quantify fragmentation of known functional elements, we computed the number of distinct predicted constrained elements overlapping each annotated coding exon. While the total number of exons that overlap at least one constrained element prediction is approximately the same between the two methods, GERP++ is significantly more effective at identifying entire exons as a single predicted CE, rather than fragmented between two or more CEs like phastCons (Fig 6C & 6D). This phenomenon is not limited to coding exons, as we observed similar behavior for experimentally identified RNA Polymerase II (PolII) binding sites (see Methods), which correspond to poised or active promoters. GERP++ overlaps a larger fraction of nucleotides within 50 base pairs of a PolII binding site (26% vs 19% for phastCons), and exhibits similarly reduced fragmentation as with coding exons (Fig 7).

Due in part to its ability to annotate larger elements in one piece, GERP++ is more effective at predicting constraint within several types of known functional regions. At the nucleotide level GERP++ elements cover a substantially larger fraction of several major types of functional elements, especially coding exons and UTRs (Fig 6B). The improved resolution in detection of known functional elements suggests GERP++ may also be more effective at predicting unannotated regions that are not only constrained but also functional.

## Discussion

One of the main challenges in constrained element detection is the lack of a clear gold standard for evaluating the quality of predictions. Human functional elements are sometimes unconstrained at the mammalian scope or missed at the assembly or alignment stages, and CE predictions that do not correspond to any known annotations may have unknown function, and cannot be definitively considered false positives. Given these limitations, we have shown that GERP++ offers several advantages over its predecessor GERP and makes fewer assumptions about the shape of conservation than previous approaches such as PhastCons. Previous bottom-up approaches have been limited largely by the simple heuristics used to merge constrained positions into longer elements; these heuristics may introduce biases in element length due to patterned constraint such as the 3-periodicity in coding exons. With GERP++ we evaluate a much richer set of candidate elements, selecting and ranking final predictions according to statistically meaningful p-values.

Despite the added computational cost at this stage, GERP++ overall is more than 100 times faster than GERP due to the speedup in rate estimation. Because GERP++ estimates a single parameter that directly translates into evolutionary rate, rather than an independent parameter for each branch of the tree, the computation is not only faster but also results in more statistically robust estimates as alignment depth increases. GERP++ takes a few days on a typical machine or a few hours on a small cluster to complete an analysis of the human genome aligned to 33 mammalian species, and can scale to virtually any reasonable genome size and alignment depth.

Our understanding of the evolutionary forces constraining sequence variation is still limited, especially in noncoding regions. This presents a challenge for generative model-based approaches, which model implicitly or explicitly the distribution of length and intensity of constrained elements and the total genomic fraction under constraint. In contrast, rate estimation and element prediction in GERP++ are largely independent procedures, and while GERP's rejected substitution metric [5] accurately quantifies constraint intensity at individual positions, any additive position-specific scoring scheme could potentially be used instead. For example, in future implementations of the GERP++ package more elaborate or context-dependent models of nucleotide evolution could be easily incorporated in order to improve position-specific evolutionary rate estimation without drastically changing the overall algorithm.

One drawback of GERP++ and other similar approaches is sensitivity to variation in and erroneous estimates of the neutral rate of substitution. Neutral rate estimates are often subject to some uncertainty and can vary depending on the methodology, alignment quality, and genomic region. To test the ability of GERP++ to tolerate a reasonable amount of error in neutral rate estimates, we repeated our analysis with the neutral tree scaled up or down by 5 or 10%. Not surprisingly, overestimating the neutral rate leads to overprediction of constraint, and vice versa. For a fixed false positive cutoff, we observed a linear relationship between the input neutral rate and the amount of constrained element bases predicted; a 5/10% change in neutral rate leads to approximately 8/15% change in the number of predicted constrained bases.

It is important to note that our false positive rates and p-values are computed based on the implicit assumption that the score distribution is homogeneous within a region and all sites are independent. While this assumption has been present in previous approaches that also relied in permuted alignments for false positive rate estimation, it is central to the GERP++ p-value computation. Finally, the greedy manner of resolving candidate element overlap conflicts by smallest p-value presents another potential limitation, as for elements with equal average constraint this will break ties in favor of the longer element. This may or may not be biologically meaningful, especially if complicated conservation patterns are involved or two strongly conserved functional elements are very close together (and the segment between them is at least somewhat constrained). These hypothetical effects are likely mitigated by GERP++'s position-specific scores, which enable higher resolution analysis within individual CEs, and which ultimately may be the criterion upon which to decide whether any particular long element may better be regarded as two shorter ones.

GERP++ recapitulates known biology, at both the nucleotide level and on the scale of entire functional elements and even chromosomes. GERP++ scores are accurate enough to obtain a strong signal of synonymous substitution in coding exons, and the elevated average RS score for chromosome X (Fig 2A) agrees with earlier findings [2], [3]. Compared to phastCons, GERP++ predictions overlap a larger fraction of known functional elements (Fig 4B) and have greater 1∶1 correspondence to constrained coding exons (Fig 6C & 6D) and promoters (Fig 7). Our analysis has also yielded interesting biological insights, including the likely presence of unannotated coding exons among our predicted constrained elements. We detect around 7% of the human genome to be contained in CEs in the mammalian scope, a slightly larger amount than previous predictions, yet with a lower estimated false positive rate. While this estimate is inexact, our analysis suggests 6% and 8% as reasonable lower and upper bounds, a somewhat tighter range than earlier estimates [1], [2].

Computationally, GERP++ is efficient enough to perform whole-genome analysis of deep mammalian alignments within a few cpu-days, making it suitable for high-throughput analysis of the ever increasing amounts of genomic data. We hope GERP++ will prove to be a useful tool in analyzing, quantifying, and annotating constraint and discovering novel functional elements in the human and other genomes for which sufficient comparative data exist.

## Methods

### Availability

GERP++ is available at http://mendel.stanford.edu/SidowLab/downloads/gerp/index.html

### Estimation of Evolutionary Rates and RS Scores

Given a multiple sequence alignment and a phylogenetic tree with branch lengths representing the neutral rate between the species within that alignment, GERP++ quantifies constraint intensity at each individual position in terms of rejected substitutions [5], the difference between the neutral rate and the estimated evolutionary rate at the position. For our analysis the alignment was compressed to remove gaps in the reference sequence (human), although the RS score computation algorithm does not assume any specific reference sequence. In order to estimate the evolutionary rate we model nucleotide evolution as a continuous-time Markov process, which specifies for each pair of nucleotides *a* and *b* and duration *t* the probability of *a* transforming into *b* over time *t*, designated by p_ab_(t). Many such evolutionary models have been developed [11], [12], each with its own set of simplifying assumptions. GERP++ implements the HKY85 model [13], but any time-reversible model (where p_a_p_ab_(t) = p_b_p_ba_(t) for all pairs of nucleotides *a* and *b*) that permits efficient computation of p_ab_(t) can be used instead.

For each individual alignment column GERP++ labels the leaves of the phylogenetic tree with the corresponding nucleotides c_1_, …, c_k_; gapped species are projected out. Although this is not necessarily ideal and sometimes leads to information loss, it avoids some of the common difficulties and potentially serious biases that accompany modeling gaps in alignments: aligner errors and artifacts that result from simplified gap penalties and incorrect handling of duplications and rearrangements, assembly mistakes, and missing sequence data. Furthermore, this treatment of gaps avoids explicitly penalizing constrained elements that have undergone lineage-specific deletion [5].

Once the gapped species are removed, the site-specific neutral rate is computed as the sum of the branch lengths in the trimmed tree. When there are fewer than 3 species remaining no rate estimation is performed for that position, as there are not enough species to even form a valid tree. We estimate by maximum likelihood a homogeneous scaling factor of the neutral tree at each position; similar but independently developed methods were used for rate estimation in [14], [15]. Specifically, we introduce a scaling parameter *r* that represents the site's rate of evolution relative to neutrality. When *r*<1 the quantity (*1−r*) can be naturally interpreted as the fraction of neutral substitutions “rejected” by evolutionary selection. GERP++ estimates *r* by maximum likelihood, where the likelihood is given by L(*r*) = Pr(c_1_, …, c_k_ | T*_r_*), where T*_r_* is the neutral tree T scaled by *r*. For any given *r*, and therefore fixed tree T*_r_*, this function can be computed efficiently using a dynamic programming algorithm due to Felsenstein [16]. If n is an internal node with children n_1_ and n_2_, and {c_1_, …, c_k_}_n_ represents the subset of the leaves corresponding to the subtree rooted at n, then
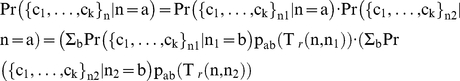
where T*_r_*(x,y) is the branch lengths in T*_r_* between two neighboring nodes x and y.

Since the leaf nucleotides are observed, this equation can be used to compute the subtree probability for all internal nodes, starting at the bottom and reaching the root, where we can compute L(*r*) = Pr(c_1_, …, c_k_ | T*_r_*) = Σ_a_ Pr({c_1_, …, c_k_}_n_ | root = a) p_a_. Assuming a fixed alphabet and an evolutionary model where the probabilities p_ab_(t) are computable in constant time, this algorithm runs in time O(k) where k is the number of species in the phylogenetic tree.

Using this algorithm as a subroutine to calculate L(*r*), GERP++ computes the maximum likelihood value of *r* using Brent's method [17], [18], a numerical optimization technique that tends to require relatively few computations of the function being optimized. The evolutionary rate for a site with neutral rate *n* is estimated to be *rn*, and the final RS score is computed as *n−rn = n(1−r)*. As maximum likelihood may estimate very large or even infinite values of *r*, we impose a cap of *r* = 3 on GERP++ rate estimates, yielding RS scores that range between *−2n* and *+n*. These scores are then used as the basis for prediction of constrained elements within the region.

### Computation of P-Values and Element Prediction

Given position-specific constraint scores, GERP++ generates a list of elements that exhibit evidence of evolutionary constraint beyond what is likely to occur by chance. For each element, we compute a p-value that represents the probability of a random neutral segment of equal length having an equal or higher RS score. In addition to being used to select final predictions from the set of candidate elements, these p-values in conjunction with position-specific scores provide useful information for biological analysis.

Every segment of contiguous multiple alignment columns is a candidate element. Because considering all possible segments within the alignment is computationally infeasible, GERP++ generates a list of candidate elements using several simple biological heuristics to prune the possibilities. First, we impose a user-specified minimum and maximum on candidate element length; while real functional elements vary in length, very few extend beyond several thousand bases, and even these will not be missed entirely as GERP++ will identify their most constrained parts. Second, since positive RS scores indicate constraint, GERP++ allows only candidate elements that start and end at positions with RS≥0 and cannot be extended further in either direction; this rule has the additional benefit of imposing sensible boundary conditions on predicted elements. Finally, we only consider candidate elements with score above a certain value, which is a function of the element length and the median neutral rate of the region. This allows pruning of candidate elements that have low scores relative to their lengths, and since they will end up with poor p-values anyway ignoring them early reduces the memory requirements considerably.

Using neutrality as the null hypothesis, we can now define p-values for candidate and predicted elements on the basis of score and length. If the probability of a single neutral position having RS score x is given by P(x), then for an element of length L and score S the p-value is the probability of having score at least S in exactly L positions, and is given by:
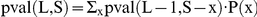
The RS score distribution is irregular (Fig S3) and therefore cannot be easily modeled by common statistical distributions; however, the p-values can be computed using dynamic programming, for L = 1, …, L_max_, provided the distribution P(x) can be computed and the space of possible scores x is not too large. The latter is assured by discretizing to within a specified tolerance t; since individual scores range from −2n to +n, there are 3n/t possible discretized scores. We now build a histogram of these discrete scores from the alignment, with two exceptions. First, we exclude long consecutive runs of “shallow” positions (default at least 10), i.e. positions with neutral rate below specified cutoff (default 0.5 substitutions per site), as there are many such primate-specific regions and they tend to skew the score distribution. Additionally, remaining shallow positions are given a small penalty to discourage GERP++ from predicting CEs consisting mostly of shallow positions. Second, we exclude positions that belong to clearly constrained regions, which are identified using a preliminary pass of the algorithm (with false positive cutoff set to 0). All other scores are used to build a score histogram for each region. In order to eliminate artifacts caused by zero probabilities, we add a small uniform prior to the histogram to ensure every discretized score appears at least once.

Once all candidate elements have been assigned p-values, GERP++ selects elements in a greedy manner, from smallest to highest p-value, discarding any elements that overlap previously reported elements. As the p-value increases so does the expected false positive rate of our predictions; when this reaches a user-specified threshold the algorithm terminates. While it would be ideal to compute this directly from the p-values, the multiple hypothesis correction in this case is non-trivial because GERP++ reports a non-overlapping set of predictions. Therefore, we adopt the approach of Cooper et al [2], [5] and estimate the false positive rate by generating several independent permuted alignments. These alignments are obtained by randomly shuffling columns of the original multiple alignments, excluding long stretches of shallow positions.

### Overview of the Data

TBA [19] alignments of the human genome (hg18) to 43 other vertebrate species were obtained from the UCSC genome browser [20], [21] together with a phylogenetic tree with the generally accepted topology (Fig S1) and neutral branch lengths estimated from 4-fold degenerate sites. Both the tree and alignments were projected to the 34 mammalian species. The alignment was compressed to remove gaps in the human sequence, and GERP++ scores were computed for every position with at least 3 ungapped species present, or approximately 88.9% of the 3.08 billion positions on the 22 autosomes and X/Y chromosomes. We used the HKY85 [13] model of evolution with the transition/transversion ratio set to 2.0 and nucleotide frequencies estimated from the multiple alignment.

To limit memory requirements and allow parallelization of the constrained element computation, each chromosome was broken up into regions of approximately 2 megabases, with long segments where no RS score was computed chosen as boundaries. These boundary segments contain no information usable by GERP++ and because the algorithm never annotates constrained elements spanning them, excluding such segments did not sacrifice any predictive ability. These boundary regions made up approximately 6.8% of the human genome, including a 30.2 megabase region that made up more than half of chromosome Y. Constrained element predictions were generated using default parameters and a 5% false positive cutoff measured in terms of number of predictions; the estimated nucleotide-level false positive rate was under 1%. As additional validation, we computed overlap between our predictions and a set of ancestral repeats (L2) annotated by RepeatMasker. We found the overlap to be in line with what we expected given our estimated false positive rates: about 5% of the repeats overlap a predicted CE, with around 1.6% nucleotide-level overlap.

Gene, noncoding RNA, and PhastCons conserved element annotations were obtained from the UCSC genome browser's [20], [21] Known Genes [22], RNA Genes, and Conservation [4] tracks respectively. To avoid skewed statistics due to alternative splicing, gene annotations were resolved to a consistent nonoverlapping set where any segment belonging to multiple conflicting annotations was assigned a single annotation in the following order of priority: coding exon, 5′ UTR, 3′ UTR, intron. For meaningful comparison against phastCons, separate GERP++ scores and constrained elements were generated according to the same procedure as above but using only placental mammal data (ignoring platypus and opossum in the alignment and projecting them out of the phylogenetic tree).

PolII binding regions were defined as 50 bp upstream and downstream of PolII binding ‘peaks’ as identified from ChIP-seq experiments performed by the ENCODE Consortium [3]. A 100 bp window allows capture of the likely PolII binding site and its flanking sequence. We obtained data from nine ChIP-seq experiments conducted in two labs (the Snyder lab at Yale and the Myers lab at Hudson Alpha) on six cell types. Data was downloaded through the DCC at UCSC (ftp://encodeftp.cse.ucsc.edu). All data have passed publication embargo periods. Overlap statistics were calculated as described above for other annotation sets and averaged across all nine experiments.

## Supporting Information

Figure S1 Phylogenetic tree used for GERP++ analysis. Tree is drawn to scale with respect to estimated neutral branch lengths.
(0.12 MB PDF)

Figure S2 Distribution of constrained element lengths. (A) GERP++. (B) PhastCons.
(0.15 MB PDF)

Figure S3 Distribution of GERP++ RS scores for 2Mb region of chromosome 1, excluding shallow (neutral rate<0.5) positions.
(0.01 MB PDF)
