# Supplementary figures and images for: Identifying a High Fraction of the Human Genome to be under Selective Constraint Using GERP++
Source: PLoS Comput Biol. 2010 Dec 2;6(12):e1001025. doi: 10.1371/journal.pcbi.1001025 (PMC2996323; doi:10.1371/journal.pcbi.1001025)

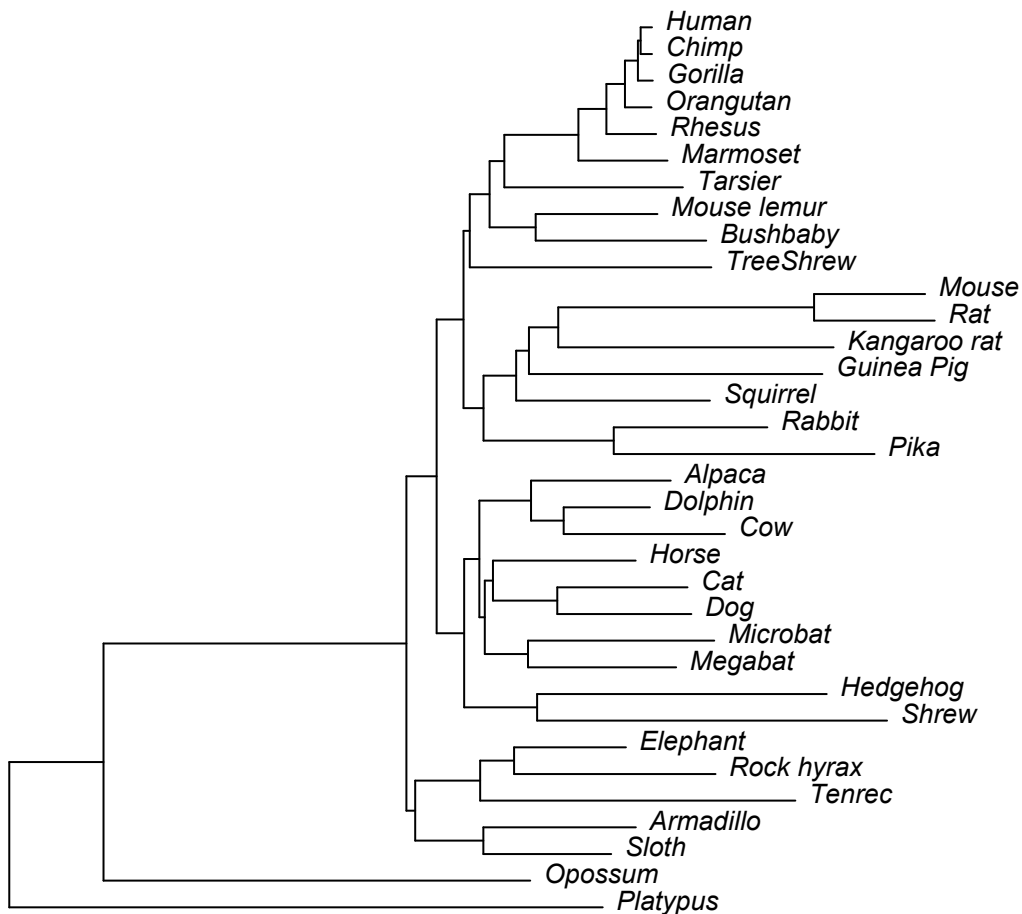

Supplement: Figure S1 — Phylogenetic tree used for GERP++ analysis. Tree is drawn to scale with respect to estimated neutral branch lengths. (0.12 MB PDF) [file pcbi.1001025.s001.pdf]

**A****Distribution of GERP++ Element Lengths**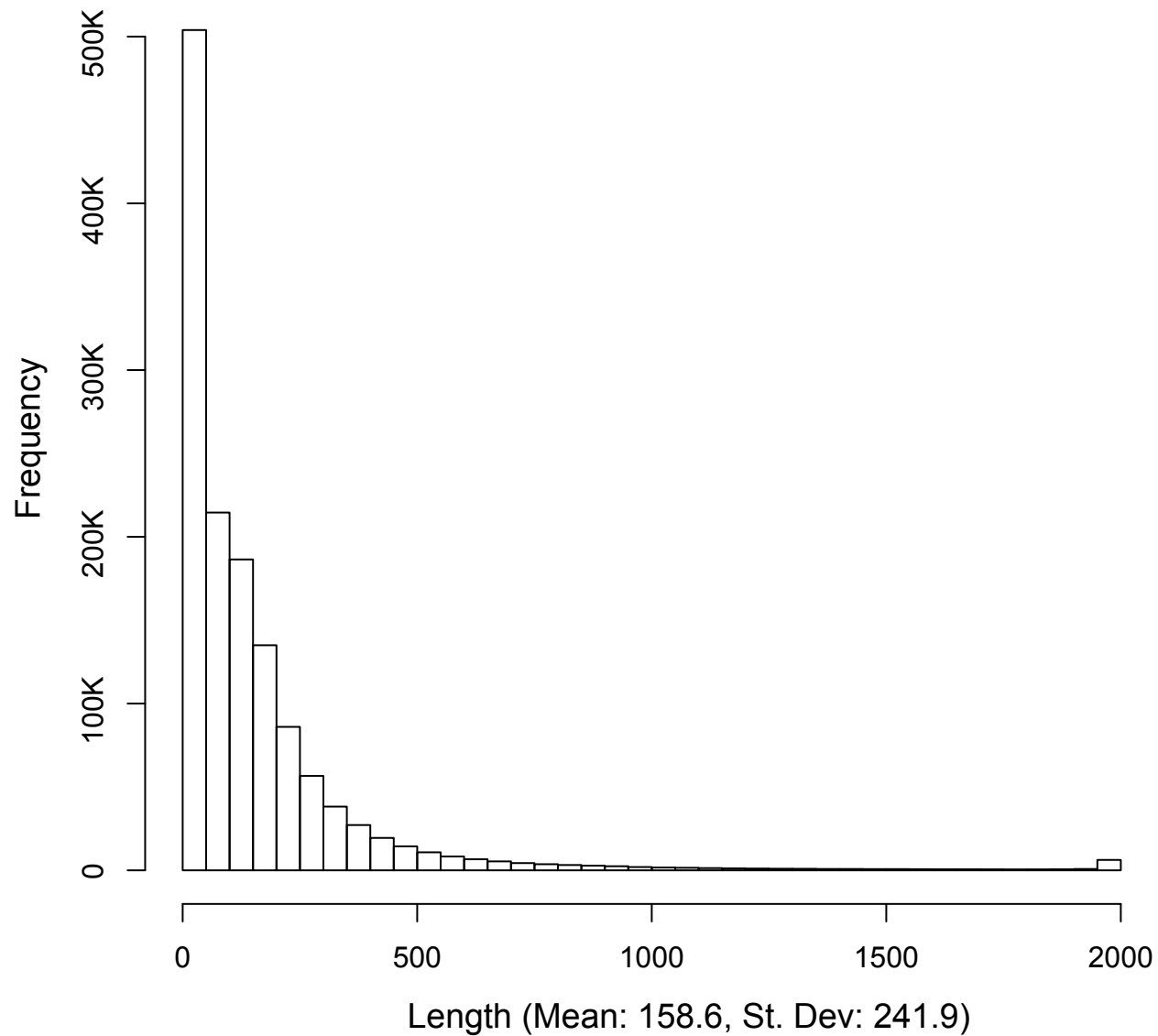**B****Distribution of PhastCons Element Lengths**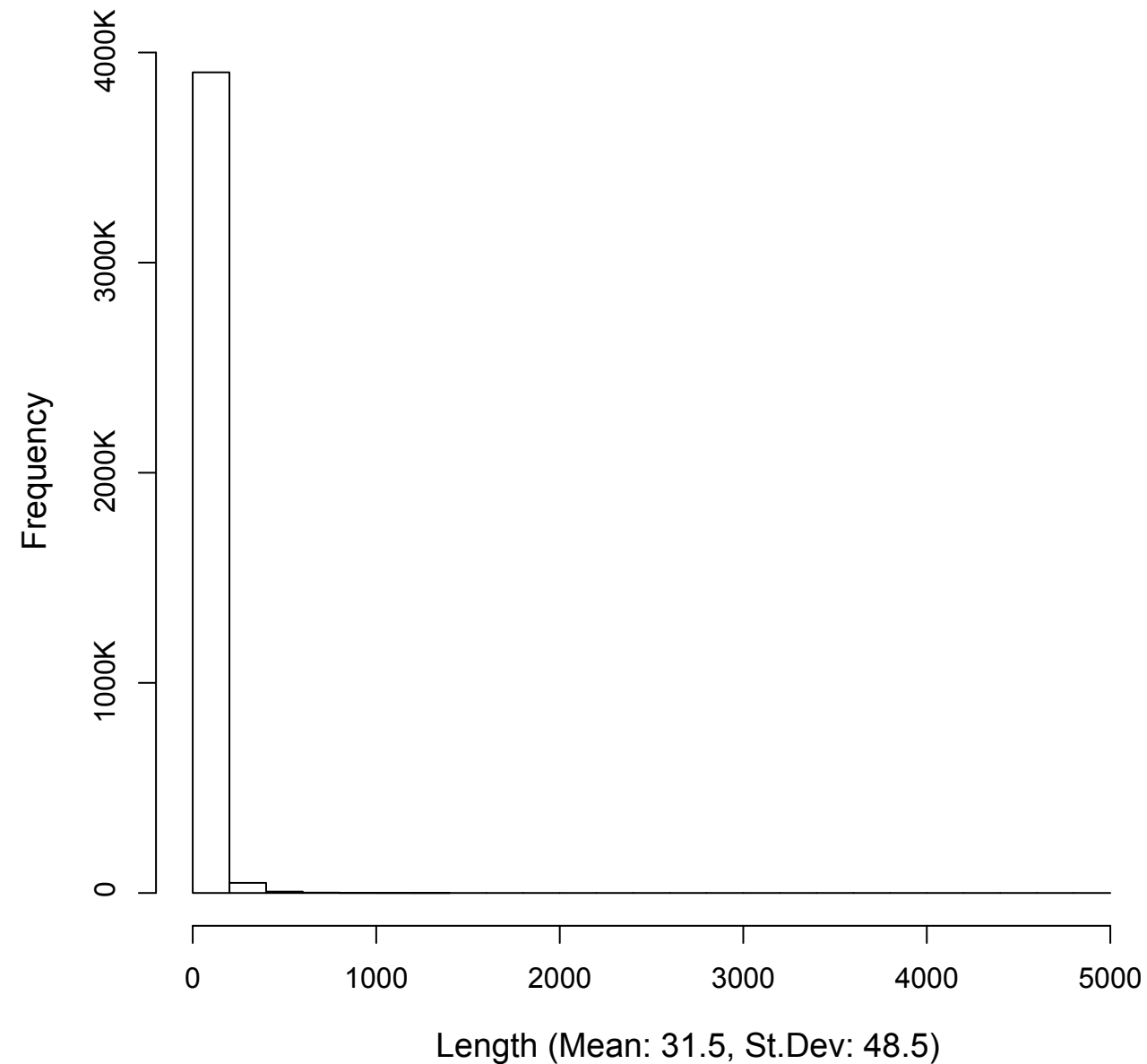

Supplement: Figure S2 — Distribution of constrained element lengths. (A) GERP++. (B) PhastCons. (0.15 MB PDF) [file pcbi.1001025.s002.pdf]

# Histogram of RS Scores

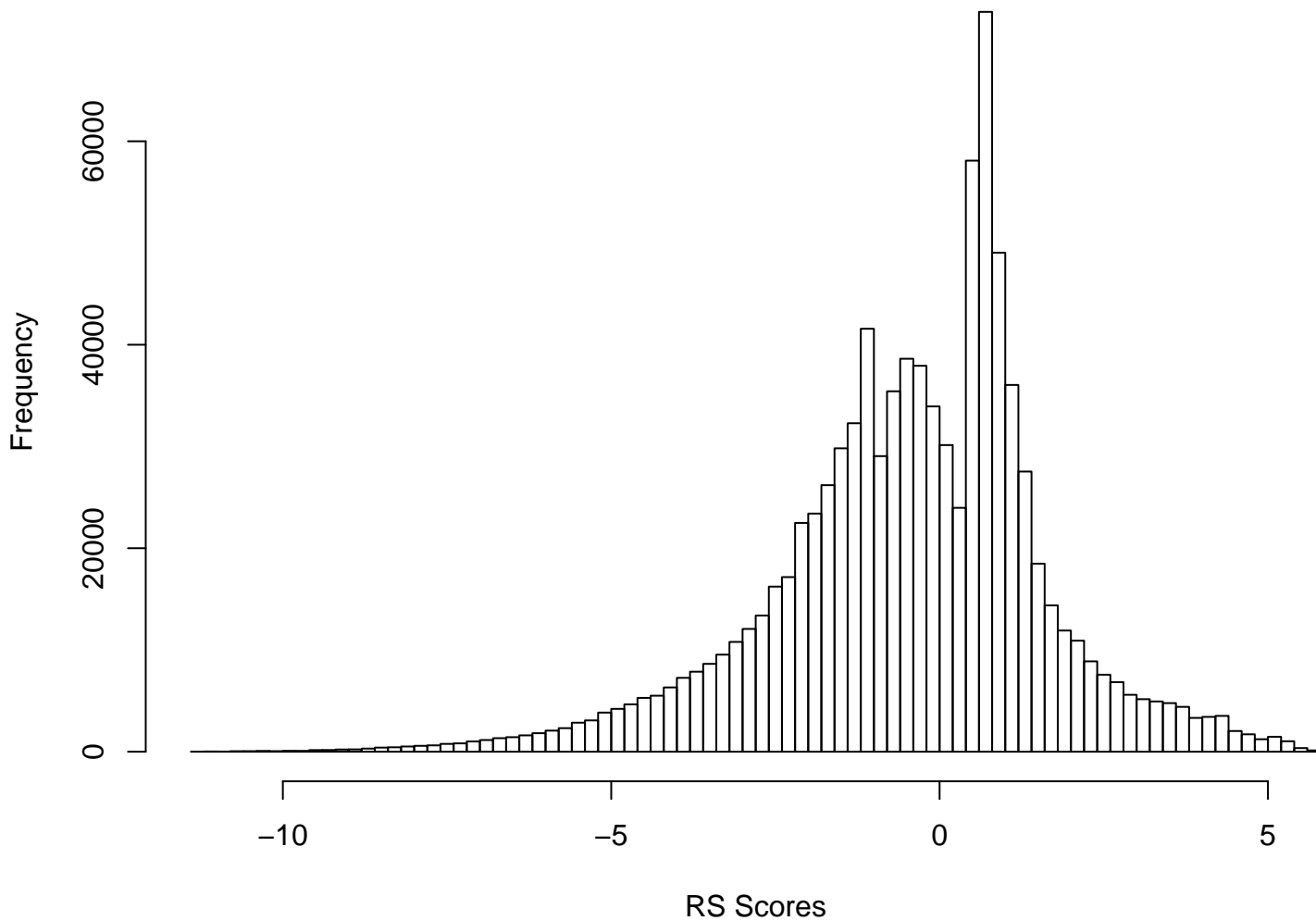

Supplement: Figure S3 — Distribution of GERP++ RS scores for 2Mb region of chromosome 1, excluding shallow (neutral rate<0.5) positions. (0.01 MB PDF) [file pcbi.1001025.s003.pdf]
